# Uptake and outcomes of early infant male circumcision services in four counties in Western Kenya

**DOI:** 10.4314/ahs.v21i1.10S

**Published:** 2021-05

**Authors:** Thomas Okwaro Andale, Onesmus Gachuno, Theresa Odero Mary Awuor

**Affiliations:** 1 School of Medicine, Moi University, and Centre for Disease Control and Prevention, Global Division for HIV and Tuberculosis (CDC/DGHT) Program Western Kenya; 2 School of Medicine, University of Nairobi

**Keywords:** Adverse event, circumcision, early male infant, uptake, western Kenya

## Abstract

**Background:**

Early Infant Male Circumcision (EIMC) is part of sustainable HIV prevention strategies in Kenya. The goals of the national EIMC program are to circumcise at least 40% of all newborn male infants delivered at hospitals offering the service and keep the rate of moderate and adverse events below 2%.

**Objectives:**

To determine the proportion of early male infants (age less than 60 days) born at hospitals in four counties of western Kenya who got circumcised and document the prevalence of adverse events (AEs) among those circumcised.

**Methods:**

A retrospective descriptive study involving all records for EIMC from 1st March 2014 through 31st March 2018 in four counties of western Kenya. Data analysis was done using EXEL to document proportion of facilities offering EIMC and compare EIMC uptake and outcomes in the four counties against the national goals for the program.

**Results:**

A mean of 4.3% of total health facilities offer EIMC in the region. Siaya had the highest proportion of facilities offering EIMC while Migori had the lowest proportion. Uptake of EIMC was low at 17.4% for all male infants born, far less than the anticipated target of 40%. Average adverse event rates were 0.3%.

**Conclusion:**

EIMC uptake remains low in this region of Kenya due to small number of health facilities offering the service. The proportion of circumcised early male infants born at the target health facilities is below the national target of 40% even though the rate of adverse events among those circumcised is acceptable.

## Introduction

Voluntary Male Medical Circumcision (VMMC) was introduced as a component of the national HIV prevention program in Kenya in the year 2008 after three randomized controlled trials in African men demonstrated that male circumcision reduces the risk of HIV acquisition by approximately 60%^1^, ^2^, ^3^. Several observational studies had also found a significant correlation between male circumcision and HIV infection rates^4^, ^5^, ^6^, ^7^, ^8^, ^9^. Based on the results of these three clinical trials and the accumulated evidence from the observational studies, the World Health Organization (WHO) and United Nations Action on HIV/AIDS (UNAIDS) recommended male circumcision as part of comprehensive HIV prevention package^10^,^11^,^12^. Following these recommendations, sub-Saharan African countries set aggressive goals to scale-up of male circumcision (MC) so as to witness the intervention's effectiveness at a population level^13^, ^14^. Many African countries, including Kenya accepted tese recommendations and are rapidly scaling up VMMC as part of their HIV prevention strategies^13^, ^14^. The first phase of VMMC in Kenya targeted uncircumcised men aged 15–45 years in regions with high HIV prevalence and low male circumcision rates^15^, ^16^. Following this VMMC rollout and in effort to come up with sustainable models for VMMC, United Nations Children's Fund (UNICEF) and World Health Organization (WHO) endorsed the Early Infant Male Circumcision (EIMC), which prompted Kenya to start piloting this program in 2014 before to progressively rolling it out across the country^17^, ^18^. This second phase of Kenya's phased approach to rolling out male circumcision (MC) for HIV prevention (from 2014–2019) prioritizes EIMC for long term HIV prevention. The long-term aspiration of EIMC in Kenya was to implement the service in all Maternal-Newborn-Child Health (MNCH) facilities in the high priority VMMC counties of Nyanza region of western Kenya so as to eventually make male circumcision the norm in all Kenyan communities in this region, regardless of cultural background^17^, ^18^, ^19^. Prior pilot studies conducted by Nyanza Reproductive Health Services (NRHS) in the focus Nyanza region of western Kenya (under the banners of “mtoto msafi 1 and mtoto msafi 2”) to assess safety and acceptability of EIMC showed that EIMC is safe and acceptable in the region^19^. A similar study showing high acceptability of EIMC was done in Botswana in 2013 20. Based on these results, EIMC services were then gradually rolled out in four high-priority counties of western Kenya as an integral component of Maternal, Newborn Child Health (MNCH) services^18^. These focal counties are Siaya, Kisumu, Homabay and Migori counties and the inhabitants of these counties are the “Luo” community.

The aim of EIMC program in Kenya was to circumcise at least 40% of all early male infants who come into contact with EIMC providing facilities within 60 days after birth by 2019^18^. EIMC service delivery targets outlined in the program's strategic plan indicated that by the end of the program phase (in 2019), each site that initiated EIMC service delivery should have circumcised at least 40% of early male infants born at or who come into contact with the facility within 60 days after birth^18^. The strategic plan further recommended the rate of Adverse Events (AEs) from all circumcision-related procedures to be kept at less than 2%^18^. The initiation of this phase of EIMC started in the four high-priority VMMC counties of western Kenya (Siaya, Kisumu, Homabay and Migori). The implementation of EIMC in these counties was supported by the Center for Disease Control and Prevention (CDC)-Kenya implementation partners. These partners were the Center for Health Solutions, CHS (for Siaya County, University of California San Francisco, UCSF (for Kisumu County), Elizabeth Glazier Pediatric AIDS Foundation, EGPAF (for Homabay County) and the University of Maryland Baltimore, UMB (for Migori County)^18^.

The aim of this study was thus to describe the uptake and outcomes of EIMC services in the four high-priority VMMC counties of western Kenya. The study mainly focused on the male infants born within the counties' health care facilities in the four counties who return for EIMC within 60 days after birth. The results of the study were compared against national program set targets as outlined in the Second Kenyan VMMC strategic plan (2014–2019)^18^. The study findings will provide a framework for addressing the likely barriers to EIMC uptake as well as offer guidance into policy guidelines for EIMC roll out in other parts of Kenya. The study was implemented after obtaining permission from EIMC program managers and ethical approval from institutional ethical review board at Moi University in western Kenya.

## Study methods

### Study site

Study was conducted in four (4) counties of western Kenya, which were classified as high priority because of high HIV prevalence and inhabited by traditionally non-circumcising community. These counties are affiliated to the CDC western Kenya branch office.

### Study population

The target group was male infants born at the health care facilities in four counties and received EIMC within 60 days of birth at the EIMC accredited facilities within the four counties. Records of clients (male infants) who were born at the EIMC accredited facilities in the four counties during a 4-year period.

### Inclusion/exclusion criteria

The study included all male infants who received EIMC at 60 days after birth. Excluded were all male infants who received EIMC after 60 days of age, those with incomplete records (i.e. missing data) plus those born at home as well as outside the health care facilities in the four counties.

### Data collection methods

Data was collected by review of EIMC records from March 2014 up to March 2018. The study had two sets of records 1) the total health facilities in each of the four counties plus the total accredited facilities certified to offer EIMC in these counties during the four-year period, and 2) EIMC data in the four counties for four years. Data was collected by two trained research assistants (RAs), one covering two counties. Each RA identified the relevant data sets for EIMC for their counties and obtained relevant data on a standard structured data collection sheet. The data was then forwarded via e-mail to the statistician at CDC office in Kisumu for entry, cleaning and analysis.

### Data analysis

Collected data was cleaned, coded and entered into an EXEL sheets for data analysis. The study variables of interest included:

#### Dependent variables

- Number of male infants born at the EIMC target facilities in the four counties
- Number of male infants (aged less than 60 days) accessing immunization services who got circumcised
- Number of early male infants circumcised who developed AEs

#### Independent variables

- Total number of health care facilities in each of the four counties
- Number of health facilities offering EIMC in each of the four counties
- Year of circumcision (2014, 2015, 2016, 2017, 2018)

Percentages and proportions of health care facilities offering EIMC against total health care facilities in each county were determined. In addition, proportions of early male infants circumcised against total number of early male infants born at the EIMC target facilities were determined. The values generated were measured against the national strategic objective targets of circumcising at least 40% of all male infants born at the EIMC providing facilities within 60 days after birth. The rate of occurrence of AEs was determined by summing up all the reported cases of AEs against the total number of circumcisions performed. A table and graphs were generated to show the trends in uptake and outcomes of the various EIMC interventions in the four counties over the 4-year period.

### Ethical considerations

The study was approved by institutional ethical research committee (IREC) at Moi University and permission to review ministry of health data base obtained from EIMC implementing partners before data collection began. The study did not have informed consent processes since there was no direct contact with patients and personally identifiable information was de-identified before analysis. It was a less than minimal risk study, the only possibility being breach of confidential patient information, and so utmost care was taken to safeguard privacy and anonymity of client data. Data sharing was only among the study team members plus the sponsors of research.

## Results

The study sample was the total number of male infants born at the healthcare facilities in the four counties of western Kenya over a four-year period. Their mean age at the time of EIMC was 22.6 days. Table 1 below is a summary of the proportion of total facilities offering EIMC, number of male infants born in all the health care facilities and the proportion of those male infants who received EIMC within 60 days of birth.

**Table 1:** EIMC Uptake/Outcome Summary for 4 Counties in Western Kenya (2014–2018)

	Year	Total Facilities	Number and % of facilities providing EIMC n (%)	Number of Male Deliveries in the county (n)	Number and % of newborn male infants circumcised n (%)	Adverse Events associated with EIMC AEs n (%)
**Homa** **Bay**	2015–16	190	11 *(5.8)*	1770	338 *(19.1)*	0 *(0)*
	2016–17	226	11 *(4.9)*	3477	339 *(9.7)*	1 *(0.3)*
	2017–18	226	11 *(4.9)*	1857	148 *(7.9)*	2 *(1.4)*
**Siaya**	2016–17	174	12 *(6.8)*	2100	390*(18.6)*	1 *(0.26)*
	2017–18	174	12 *(6.8)*	1360	245*(18.1)*	2 *(0.82)*
**Kisumu**	2016–17	203	9 *(4.4)*	2350	521*(22.2)*	1 *(0.2)*
	2017–18	203	9 *(4.4)*	2400	632*(26.3)*	0 *(0)*
**Migori**	2017–18	213	3 *(1.4)*	255	99 *(38.8)*	1 *(1.0)*

**Total**		**816** *	**35** # **(4.3).**	**15, 569** $	**2,712** $ **(17.4)**	**8** $ **(0.3)**

* Added all facilities in 2017–18.

# Added all facilities in 2017–18.

$ Overall total for all the years.

$ Overall total for all the years.

$ Overall total for all the years.

**Figure 1:**
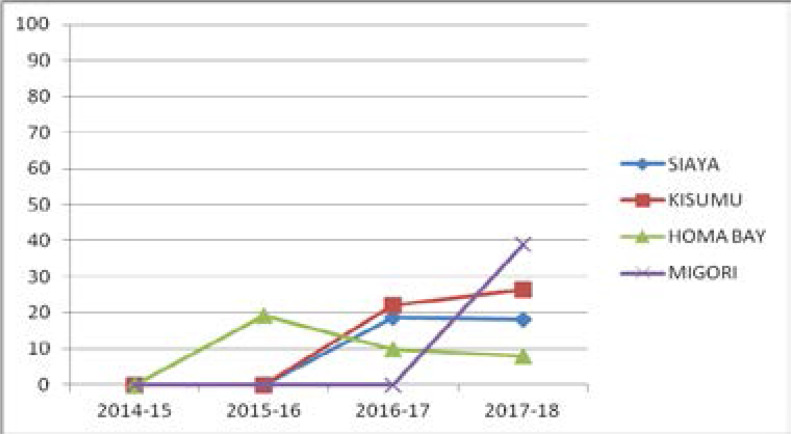
Trends in EIMC uptake in four counties from 2014–18.

In Figure 1 below, the uptake of EIMC increased in Migori and Kisumu counties while it dropped in Siaya and Homabay counties.

## Discussion

Although the Kenyan VMMC strategic plan (2014–2019) had indicated that EIMC roll-out would begin in 2014 and proceed till 2019 17,18, this did not happen and a number of factors could have led to EIMC roll-out starting at different times in all the 4 counties, with Siaya County starting its program rollout in 2016, Kisumu in 2015, Homabay in 2015 and Migori in 2017. No county started program roll-out on time as stipulated in the program targets. Some of the postulated reasons that delayed the roll-out could include lack of adequate funds for implementing the EIMC program, which led to delays in certifying EIMC facilities, as well as delays in training staffs on EIMC^21^, ^22^. Another reason could be the slow integration of EIMC into the routine maternal-neonatal-child health (MNCH) services, which significantly impacted rollout in all these four counties^21^,^22^.

According to Table 1, the number of male infants offered EIMC represents between 7.9–19.1% of total male infants born at the health facilities in this region. This figure is still very low when compared to the set national target of offering EIMC to at least 40% of all male infant hospital deliveries. A number of factors could be attributed to this low uptake of EIMC in this region. First of all, previous studies had indicated that the lack of circumcision as a cultural tradition in this region is a major barrier that needs to be dealt with during community sensitizations^19^. The native inhabitants of these four counties (the Luo community) are traditionally a non-circumcising community and hence changing their attitudes and behaviors towards the practice has to take time. Fear of complications has also been cited in many studies as among the reasons why most mothers do not circumcise their sons at infancy^19^, ^23^, ^24^. These issues need to be addressed at education/counseling sessions with community members and pregnant mothers.

Another factor that could have led to low uptake of EIMC services is lack of access. The results show that the number of health facilities offering EIMC represents a very small proportion of the total health facilities in the counties (range=4.9–5.8%). The trend in EIMC uptake also showed that this number of health facilities offering EIMC remained fairly constant in all the counties during the study period and there were few new facilities being upgraded and accredited to offer EIMC from 2015 to 2018. These reasons could have contributed to the low uptake of EIMC services in this region^19^, ^23^. A lot therefore still remains to be done to accredit more health facilities in all the four counties. On the other hand, the AEs remained at 0.3% for the total EIMC in all the four counties over the study period. The national target is to keep AEs arising from EIMC at less than 2%^19^.

## Conclusion

The EIMC uptake in this program is still low against the expected program target, even though the rate of AEs among those who received EIMC is below the expected cut-off of 2%. More efforts, therefore, have to be put in place to enhance uptake of this service. The study had a number of limitations. First of all, this being a retrospective study, there was the inherent weakness of missing and/or incomplete data. Therefore, all patient records with missing data were excluded and data analysis was adjusted according to completeness of information obtained.
